# Chin tremor in full-term neonate after hypoxia

**DOI:** 10.1590/S1516-31802012000600009

**Published:** 2013-01-18

**Authors:** Mônica Ayres de Araújo Scattolin, Catherine Marx, Ruth Guinsburg, Marcelo Rodrigues Masruha, Luiz Celso Pereira Vilanova

**Affiliations:** I MD. Child Neurologist, Department of Neurology and Neurosurgery, Universidade Federal de São Paulo (Unifesp), São Paulo, Brazil.; II MD, PhD. Professor, Department of Neonatology, Universidade Federal de São Paulo (Unifesp), São Paulo, Brazil.; III MD, PhD. Neurologist and Assistant Professor, Department of Neurology and Neurosurgery, Universidade Federal de São Paulo (Unifesp), São Paulo, Brazil.

**Keywords:** Hypoxia, brain, Tremor, Chin, Infant, newborn, Neurology, Hipóxia encefálica, Tremor, Queixo, Recém-nascido, Neurologia

## Abstract

**CONTEXT:**

Newborns may present a range of motor phenomena that are not epileptic in nature. Chin tremor is an unusual movement disorder that typically starts in early childhood and may be precipitated by stress and emotion. Its pathophysiology has not been fully elucidated.

**CASE REPORT:**

We describe a full-term newborn that, immediately after neonatal anoxia, presented body and chin tremors that were unresponsive to anti-epileptic drugs. Subsequent neurological evaluation revealed signs of pyramidal tract damage and chin tremor triggered by percussion and crying. We discuss the hypothesis that the anatomopathological abnormality may lie at the level of the higher cortical centers or midbrain.

**CONCLUSIONS:**

Further studies are needed in order to gain greater comprehension of neonatal tremors. Recognition of the various etiological possibilities and consequent management of treatable causes is essential for care optimization.

## RESUMO

**CONTEXTO:**

O recém-nascido está sujeito a uma gama de fenômenos motores de natureza não epiléptica. O tremor do mento é um distúrbio do movimento incomum que tem início habitual na infância e pode ser precipitado por estresse e emoção. Sua fisiopatologia não foi completamente elucidada.

**RELATO DE CASO:**

Descrevemos um recém-nascido no termo, que, após anóxia neonatal, apresentou tremor de corpo e mento não responsivo ao uso de drogas antiepilépticas. A avaliação neurológica posterior revelou sinais de lesão do trato piramidal e tremor de mento desencadeado por choro e percussão. Discutimos a hipótese de que as alterações anatomopatológicas estejam localizadas no mesencéfalo ou centros corticais superiores.

**CONCLUSÕES:**

São necessários novos estudos para maior compreensão dos tremores em recém-nascidos. O reconhecimento das diversas possibilidades etiológicas e o decorrente manejo das causas tratáveis são essenciais para a otimização do atendimento.

## INTRODUCTION

Newborns may present a range of clinical motor phenomena that are not epileptic in nature. Tremors are one of them and they are generally underrated. They consist of a rhythmic back-and-forth or oscillating involuntary movement about a joint axis.^1^

Two thirds of healthy newborns exhibit mild tremors during the first few days of life.^2^ The neurological outcome among infants with no neurological signs except for tremors seems to be good, especially when there are no perinatal complications. Infants with these complications, however, should be monitored closely because about 30% of the patients with complications may present neurological deficits, such as borderline intelligence or mild cerebral palsy. These deficits are thought to be related to underlying brain damage caused by perinatal injuries.^3^

In a recent prospective study on 103 patients with ages ranging from two to 70 years, the prevalence of tremors in patients with secondary movement disorders (SMD) was found to be 12.6%. The tremors involved the right upper limb in 69.2%, left upper limb in 15.4%, head in 7.7% and generalized distribution in 7.7% patients. The etiologies included space-occupying lesions in eight patients (61.5%), trauma in four (30.8%) and vascular lesions in one (7.7%). The study highlighted the lack of correlation between types of SMD and underlying etiologies.^4^

## CASE REPORT

After a 40-week uncomplicated pregnancy, a male infant weighing 3,460 grams was born from spontaneous vaginal delivery to a mother with controlled hypothyroidism (consequent to treated Graves’ disease that had been diagnosed five years earlier). The parents were first-degree cousins. The baby was admitted to the maternity ward of São Paulo Hospital, which is a tertiary-level hospital in São Paulo, Brazil.

The one-minute and five-minute Apgar scores were 2 and 7, respectively. On his first examination, there were no dysmorphic features in the baby and the head circumference was normal.

The patient fulfilled all the encephalopathy criteria for therapeutic hypothermia (arterial blood cord pH: 6.87; base deficit: -23.6 mmol/l; hypotonia; and requirement for mechanical ventilation assistance). Administration of 72 hours of whole-body therapeutic hypothermia with a core temperature of 33.5-34.5°C commenced within the first hour of life. During this 72-hour intervention period, the infant presented an episode of hyperglycemia and hypotension, which was treated with vasopressors.

**Figure 1.**
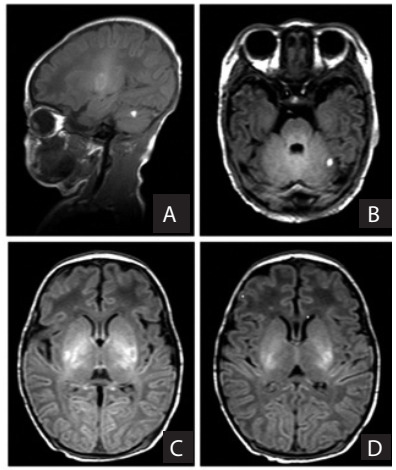
Non-enhanced sagittal (A) and axial (B, C, D) magnetic resonance imaging: abnormally increased signal intensities relating to cerebellar hemorrhage (A) and (B); and bilateral lentiform nucleus and thalamic presence of methemoglobin (C) and (D), as a consequence of hemorrhagic transformation after ischemic injury.

Body and chin tremors were noted within the first hour of life and were initially interpreted as seizures by the neonatal staff. A neurological consultation was obtained after it was observed that the patient had no response to optimized antiepileptic drug (phenobarbital). There was no familial history of tremors. An electroencephalogram (EEG) using the 10-20 international conventional montage was performed at 72 hours of life and registered abnormal and diffusely slow base activity. A magnetic resonance imaging (MRI) examination at seven days of life showed signs of cerebellar hemorrhage and bilateral thalamic ischemia **(**Figure 1**)**.

Twenty-three days later, his EEG during wakefulness was normal. During these events, no epileptiform activity was observed. A neurological evaluation after normal temperature had been achieved revealed signs of pyramidal tract damage, and chin tremors triggered by percussion and crying. Tone was diffusely and symmetrically increased; deep tendon reflexes were increased but symmetrical; and sustained ankle clonus was present.

The chin tremor condition diminished despite antiepileptic drug use. The baby was discharged at the age of seven days and was referred for multidisciplinary follow-up. At clinical care consultations, the mother reported that her child was difficult to console and presented sporadic chin tremors during intense crying. The child is currently five months old and his development is mildly delayed.

## DISCUSSION

There are several possible etiologies for the patient’s chin tremor. We carried out a systematic analysis of the published indexed articles, using the combined strategy (anoxia OR hypoxia) AND newborn AND tremor. Additional relevant reports were obtained by using the terms neonatal jaw clonus, geniospasm, infant jaw clonus, infant jaw movements, mandibular oscillation and chin trembling.

The online databases searched were: Lilacs (Literatura Latino-Americana e do Caribe em Ciências da Saúde), Cochrane Library, Excerpta Medica Database (Embase) and Medline PubMed **(**Table 1**)**.

**Table 1.** Search for case reports, in Medline (Medical Literature Analysis and Retrieval System Online), Excerpta Medica Database (Embase), Cochrane Library and Lilacs (Literatura Latino-Americana e do Caribe em Ciências da Saúde) databases, done on July 26, 2011

Database	Search strategies	Results
Lilacs*	(anoxia OR hypoxia) AND newborn AND tremor	Papers found: 1 Papers related: 1
Embase	(anoxia OR hypoxia) AND newborn AND tremor	Papers found: 3 Papers related: 2
Cochrane Library	(anoxia OR hypoxia) AND newborn AND tremor	Papers found: 2 Papers related: 0
Medline	(anoxia OR hypoxia) AND newborn AND tremor	Papers found: 4 Papers related: 1

*Results achieved with the terms in Spanish and Portuguese were also found within the search strategy in English.

**Chart 1.**
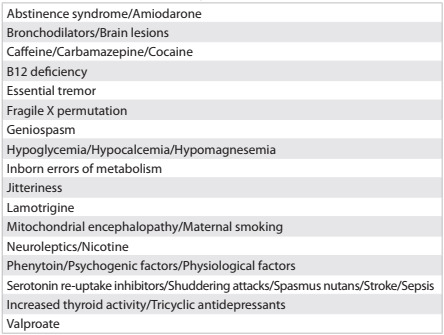
Neonatal tremor etiologies

The pathological conditions that may be associated with tremor include hypoglycemia, hypoxic ischemic encephalopathy, intracranial hemorrhage, hypothermia, hyperthyroid state and drug withdrawal **(**Chart 1**)**. The patient was investigated for a hyperthyroid state, since his mother had had Graves’ disease. Transient neonatal hyperthyroidism has been reported in 1-5% of babies born from mothers with Graves’ disease, due to transplacental passage of the maternal immunoglobulin.^5^ The risk of neonatal thyrotoxicosis is highest in infants exposed to high maternal immunoglobulin titers during the last trimester of pregnancy.^6^ These previous findings prompted investigation, which showed normal thyroid-stimulating hormone and free thyroxine, thereby ruling out such possibilities.

In our case, the tremors occurred both before and after hypothermic neural rescue therapy. Since electrographic seizures are very frequent in neonates during head cooling and often lack a clinical correlate, an EEG was performed to rule out this possibility. Tremors can be clinically differentiated from seizures in that they can be brought on with stimuli and can be stopped with gentle passive flexion and restraint of the affected limb. They are not associated with ocular phenomena, such as eye deviation or with significant autonomic changes such as tachycardia or apnea.^7^

The presence of tremors after hypothermia therapy has been little discussed. The medulla oblongata has been found to be a structure that is sensitive to temperature changes, and cooling of this structure in monkeys was found to induce shivering of the jaws.^8^ We can only speculate whether the hypothermia contributed towards this patient’s clinical presentation.

The physiopathology of chin tremors has not been fully elucidated.^9^ Tremors might be due to lesions within the cerebellum, cerebral cortex, basal ganglia, red nucleus or thalamus.^3^^,^^10^

One postulated mechanism is that respiratory failure leading to hypoxia can result in tremors that are essentially an enhanced 8-12 Hz physiological tremor.^11^

Although literature on tremors and hypoxia is scarce, it has been accepted that hypoxia promotes cortical arousal through activation of chemoreceptors of the ascending reticular activating system. The effects of hypoxia mainly result from a cascade of events starting with activation of the hypothalamic-pituitary-adrenal axis. In turn, this causes an increase in catecholamine release, thus leading to augmented tremor amplitude in the 6 to 12-Hz interval and heart rate increase.^12^

Another theory states that neonatal tremors are due to immaturity of the spinal inhibitory interneurons, causing an excessive muscle stretch reflex. As the neonate gets older and the interneurons mature, the tremors cease.^13^

In fact, it is generally accepted that both the central and the peripheral nervous systems contribute towards generation of physiological tremors, at proportions that vary according to the environmental conditions and the subject’s health.^12^

The term “chin trembling” yielded reports of a rare hereditary disorder characterized by involuntary trembling of the chin, also known as hereditary geniospasm. This disorder was first reported in 1922.^14^ Involuntary tremors of the chin and lower lip were observed as early as a few hours after birth and may have been precipitated by stress, concentration and emotion. Reports of male-to-male transmission^15^ suggest that there is an autosomal dominant inheritance pattern, with one locus on chromosome 9q13.^16^ Although an association with other neurological conditions, such as nystagmus,^17^ strabismus^18^ and premature hearing loss (over age 50)^19^ has been described, most of these appear to be incidental associations.

Chin tremor symmetrically affects both sides of the mentalis muscle fibers.^20^ The bilateral nature of the movement suggests that the abnormality may lie either within the muscle itself or at the level of the higher cortical or midbrain centers.

The treatment for neonates with tremors should aim to correct the underlying cause, if identified. Use of botulinum toxin injections into the perioral muscle is reserved only for cases in which the trembling causes difficulty with eating, drinking or social embarrassment.^21^

## CONCLUSION

Further studies are needed in order to gain greater comprehension of neonatal tremors. Recognition of the various etiological possibilities and consequent management of treatable causes is essential for care optimization.

## References

[1] Sanger TD, Chen D, Fehlings DL (2010). Definition and classification of hyperkinetic movements in childhood. Mov Disord.

[2] Armentrout DC, Caple J (2001). The jittery newborn. J Pediatr Health Care.

[3] Futagi Y, Suzuki Y, Toribe Y, Kato T (1999). Neurologic outcomes of infants with tremor within the first year of life. Pediatr Neurol.

[4] Netravathi M, Pal PK, Indira Devi B (2011). A clinical profile of 103 patients with secondary movement disorders: correlation of etiology with phenomenology. Eur J Neurol.

[5] Polak M (1998). Hyperthyroidism in early infancy: pathogenesis, clinical features and diagnosis with a focus on neonatal hyperthyroidism. Thyroid.

[6] Markham LA, Stevens DL (2003). A case report of neonatal thyrotoxicosis due to maternal autoimmune hyperthyroidism. Adv Neonatal Care.

[7] Volpe JJ, Volpe JJ (2001). Neonatal Seizures. Neurology of the newborn.

[8] Chai CY, Lin MT (1972). Effects of heating and cooling the spinal cord and medulla oblongata on thermoregulation in monkeys. J Physiol.

[9] Kharraz B, Reilich P, Noachtar S, Danek A (2008). An episode of geniospasm in sleep: toward new insights into pathophysiology?. Mov Disord.

[10] Sanger TD (2003). Pediatric movement disorders. Curr Opin Neurol.

[11] Krause WL, Leiter JC, Marsh Tenney S, Daubenspeck JA (2000). Acute hypoxia activates human 8-12 Hz physiological tremor. Respir Physiol.

[12] Legros A, Marshall HR, Beuter A (2010). Effects of acute hypoxia on postural and kinetic tremor. Eur J Appl Physiol.

[13] Shuper A, Zalzberg J, Weitz R, Mimouni M (1991). Jitteriness beyond the neonatal period: a benign pattern of movement in infancy. J Child Neurol.

[14] Stocks P (1923). Facial spasm inherited through four generations. Biometrika.

[15] Destee A, Cassim F, Defebvre L, Guieu JD (1997). Hereditary chin trembling or hereditary chin myoclonus?. J Neurol Neurosurg Psychiatry.

[16] Jarman PR, Wood NW, Davis MT (1997). Hereditary geniospasm: linkage to chromosome 9q13-q21 and evidence for genetic heterogeneity. Am J Hum Genet.

[17] Laurance BM, Matthews WB, Diggle JH (1968). Hereditary quivering of the chin. Arch Dis Child.

[18] Danek A (1993). Geniospasm: hereditary chin trembling. Mov Disord.

[19] Alsager DE, Bowen P, Bamforth JS (1991). Trembling chin--a report of this inheritable dominant character in a four-generation Canadian family. Clin Genet.

[20] Kharraz B, Reilich P, Noachtar S, Danek A (2008). An episode of geniospasm in sleep: toward new insights into pathophysiology?. Mov Disord.

[21] Huntsman RJ, Lowry NJ, Sanakaran K (2010). [Non epileptic motor phenomena in the newborn]. Zhongguo Dang Dai Er Ke Za Zhi.

